# Congestive Heart Failure Exacerbations and the Role of Urine Output Monitoring

**DOI:** 10.7759/cureus.93938

**Published:** 2025-10-06

**Authors:** Anthony Teta, Megan Meyers, Michael Boyle, Kerrie Kopicko, Carolina Restini, Grace D Brannan, Jay Mohan, Christopher Provenzano

**Affiliations:** 1 Cardiology, McLaren Macomb Hospital, Mount Clemens, USA; 2 Nephrology, Medical University of South Carolina, Charleston, USA; 3 Internal Medicine, Mclaren Macomb Hospital, Mount Clemens, USA; 4 Internal Medicine, Michigan State University College of Osteopathic Medicine, East Lansing, USA; 5 Pharmacology, Michigan State University College of Osteopathic Medicine, Macomb, USA; 6 Graduate Medical Education, McLaren Macomb Hospital, Athens, USA; 7 Cardiology, Mclaren Macomb Hospital, Mount Clemens, USA; 8 Nephrology, McLaren Macomb Hospital, Mount Clemens, USA

**Keywords:** diuresis, heart failure exacerbation, length of hospital stay (los), urine output, urine output monitoring

## Abstract

Background

Monitoring fluid status is considered the standard of care for many patients treated in the intensive care unit (ICU), but there is little data to support its utility outside of the ICU. Data linking urine output (UO) monitoring with shortened length of stay (LOS), specifically in non-ICU heart failure (HF) patients, is scarce. This study aimed to determine the relationship between the electronic medical record order to "monitor intake/output" and LOS in patients with HF exacerbation, the impact of consulting nephrology, and whether the timing of consultation would improve measured outcomes.

Methods

A hundred and twelve patient records with a diagnosis of “exacerbated heart failure”, including International Classification of Diseases (ICD) 10 codes of I50.23 (acute on chronic systolic HF), I50.33 (acute on chronic diastolic HF), I50.21 (acute systolic HF), I50.31 (acute diastolic HF), I50.43 (acute on chronic combined systolic and diastolic HF), and I50.41 (acute combined systolic and diastolic HF) from McLaren Macomb Hospital, Mount Clemens, Michigan were reviewed, and the following clinical outcomes were evaluated: presence of physician order to “monitor intake/output” within the electronic medical record, comorbidities, stage of chronic kidney disease (CKD), grade of diastolic dysfunction, New York Heart Association stages of HF, types of diuretics used, LOS, readmission within 30 days, follow-up, frequency of urine monitoring per patient, and if there was a nephrology consult at the time of presentation.

Results

Seventy-one (63.4%) patients received urine monitoring every eight hours, and there were no statistically significant differences between physician-ordered monitoring and comorbidities, stages of CKD, diastolic dysfunction grade, LOS, or readmission rates. The mean LOS was slightly higher in patients with the physician's order.

Conclusions

Neither UO monitoring nor consulting nephrology was shown to significantly affect LOS or readmission rates in non-ICU patients with exacerbation of HF. Different methods in estimating volume status (e.g., daily body weight measurements, noninvasive monitoring devices) may substantially affect outcomes, and future studies are needed.

## Introduction

Heart failure (HF) represents a staggering clinical and public health burden to 6.7 million Americans, and the number is projected to increase to 8.5 million by 2030. Globally, more than 64 million people live with HF [1,2]. The annual cost burden of HF is an estimated $30.7 billion, and there is a national 30-day readmission rate of 23% for Medicare patients [3-4]. Despite the progress in reducing HF-related mortality, HF hospitalizations remain frequent, and readmission rates continue to rise [5-7]. New strategies are being sought to improve patient outcomes while reducing the financial implications of HF to allow for more personalized treatment plans. For example, natriuresis-guided diuretic protocols have been shown to result in higher levels of natriuresis with shortened hospital stays [8]. Fluid retention and congestion are hallmarks of HF that worsen clinical symptoms and contribute to the development of acute kidney injury (AKI) [9-14]. A pillar of HF therapy is alleviating sodium and fluid retention with diuretics [10, 11]. Current American Heart Association (AHA), American College of Cardiology (ACC), and Heart Failure Society of America (HFSA) guidelines recommend measurement of fluid intake and output as well as assessing clinical signs of congestion in patients hospitalized with exacerbations of HF [9]. Monitoring fluid status in HF patients is also considered the standard of care for those treated for HF exacerbations in the intensive care unit (ICU) [15]. 

Prolonged fluid accumulation and positive fluid balance in ICU patients have been shown to delay patient recovery and increase mortality [16, 17]. Recording body weight changes to estimate body fluid status in ICU patients is commonly used [17]. However, previous studies have shown inaccuracy in the estimation of body weights by medical staff [18, 19]. These inaccuracies may be explained by the challenges of measuring body weight in immobilized patients who are limited to bed scales. Consequently, measurements of body weight are unreliable if not performed daily and accurately with standardized weighing procedures [17, 20]. 

Diuretics mostly used in the treatment of HF include thiazide and loop diuretics, with evidence shown for increased effectiveness of diuresis and decongestion with the addition of sequential nephron blockade using additional diuretics such as carbonic anhydrase inhibitors [21]. Diuretic doses have been determined to be strong predictors of urine output (UO) [22]. The utility of monitoring UO in the ICU setting has been well delineated in the literature and is often used as a marker of renal function [13, 23]. Monitoring UO in ICU patients is associated with increased survival rates, improved detection of AKI, and a shorter length of stay (LOS) in the ICU [23, 24]. In patients without AKI, close UO monitoring resulted in decreased cumulative fluid volume [23]. Management guidelines support monitoring UO in ICU patients because UO is an important risk assessment tool used to predict patients at greatest risk for developing an AKI [25]. However, there is little to be found regarding the significance of monitoring fluid balance in those not treated in the ICU. 

Compared to patient monitoring in the ICU setting, monitoring of patients’ vital signs and UO occurs less often in other hospital settings [26]. Renal function is often overlooked in non-ICU patients due to the lack of data regarding optimal timing and frequency of UO monitoring [26]. There is a relative lack of literature focused on non-ICU patients, including those with HF. Despite HF exacerbation being a common condition outside of the ICU, data linking UO monitoring with improved outcomes or shortened LOS in non-ICU HF patients is scarce [26, 27]. The optimal frequency of renal function monitoring in HF patients on diuretics is also limited [26]. Current guidelines for treating an acute exacerbation of HF mention close monitoring of renal function, but in real-world practice, this remains poorly implemented [26, 28]. 

The primary objective of this study was to evaluate whether the physician's order to monitor intake and UO in non-ICU patients diagnosed with acute heart failure exacerbation was associated with reduced length of stay or 30-day readmission. A secondary objective was to assess whether nephrology consultation, and specifically its timing, influenced these outcomes. It also evaluated the relationship between the physician's order to monitor intake and UO, as well as other demographic variables in non-ICU patients admitted for an exacerbation of congestive HF.

## Materials and methods

This retrospective cohort study was conducted at McLaren Macomb Hospital, Mount Clemens, Michigan, to determine how often physicians monitor UO when using intravenous (IV) diuretic therapy for these patients and the relationships among clinical and demographic factors, such as age and rates of readmission. In addition, the study assessed whether a nephrology consult correlates with a shorter LOS and if earlier consults changed outcomes. The physician's order to monitor UO in this study facilitated constant monitoring throughout each patient's hospital stay. The protocols were approved by the McLaren Health Care Institutional Review Board (IRB# 2019-00071) and evaluated using de-identified medical records in statistical analysis. Our patient population consisted of men and women who were 50 years of age or older with a final diagnosis of “exacerbated heart failure." The following International Classification of Diseases (ICD) 10 codes were used to identify the patient records for review: I50.23 (acute on chronic systolic HF), I50.33 (acute on chronic diastolic HF), I50.21 (acute systolic HF), I50.31 (acute diastolic HF), I50.43 (acute on chronic combined systolic and diastolic HF), and I50.41 (acute combined systolic and diastolic HF). 

Clinical variables and demographic information were collected. Demographic information included age, gender, ethnicity, and race. The following clinical variables were collected: presence of physician order to “monitor intake/output”, comorbidities including diabetes mellitus and hypertension, stage of chronic kidney disease, grade of diastolic dysfunction, New York Heart Association (NYHA) Class (I-IV) and/or stages of heart failure, class of diuretics used, length of hospital stay (in days), readmission within 30 days, outpatient follow-up, and whether or not each patient had the physician-placed order to monitor urine output. This study was performed at a community teaching hospital, and patients were delineated between those being cared for by resident teaching service teams and private physician services. Additionally, it was recorded whether nephrology was consulted, and if so, the time of consultation in the context of each patient’s hospital course. 

Patients were excluded if they were below 50 years in age and had contraindications to IV diuresis, such as end-stage renal disease (ESRD) on hemodialysis, anuria, chronic obstructive pulmonary disease (COPD) exacerbation, discharge disposition to a subacute rehab or skilled nursing facility, or concurrent infection that could complicate their hospital stay. These infections included, but were not limited to, ICD-10 codes: N39.0, J13, J15, J18.9, J06, J22 (urinary tract infection, streptococcus pneumonia, bacterial pneumonia, unspecified pneumonia, acute upper respiratory tract infection, and acute lower respiratory tract infection, bacteremia). There were no vulnerable populations identified for recruitment in this retrospective cohort chart review. 

We generated descriptive statistics such as means, percentages, and frequencies. Chi-square test was performed to determine statistical differences between categorical variables. An independent t-test was performed to compare the means of continuous variables. Statistical significance was set at p<0.05. International Business Machines Statistical Package for the Social Sciences (SPSS) version 25 (IBM Corp., Armonk, New York, USA) statistical program was used. 

## Results

Table 1 shows patient demographics. A total of 112 non-ICU patient records were identified and reviewed during this study. The majority of the patients were female [65 (58%); p=0.089], and all patients had a final diagnosis of “exacerbated heart failure” and were between the ages of 50 to 97-years-old, with a mean age of 77.24 years [Standard Deviation (SD) 10.79]. The majority of patients in the study (107 (95.5%)) were Caucasian. Additionally, Table 1 groups the demographic characteristics of patients by whether UO monitoring was ordered. There were no statistically significant differences between the two groups based on age in years (p=0.123), gender (p=0.752), and race (p=0.872) distribution. 

**Table 1:** Patient demographics for the total population and grouped by physician ordered UO monitoring. SD: standard deviation; NA: not applicable; UO: urine output. * Chi-square test was performed to determine statistical differences between categorical variables. An independent t-test was performed to compare the means of continuous variables. **  No p-value was calculated for physician-ordered monitoring because ethnicity is a constant.

Variable*	Total Population		Physician Ordered UO Monitoring		
	Statistic	P-Value	No	Yes	P-Value
Mean Age in Years (SD)	77.24 (10.793)	NA	75.17 (11.87)	78.44 (10.02)	0.123
Gender, N (%)					
Male	47 (42)	0.089	18 (16.1)	29 (25.9)	0.752
Female	65 (58)		23 (20.5)	42 (37.5)	
Ethnicity, N (%)					
Not Hispanic or Latino**	112 (100)	NA	41 (36.6)	71 (63.4)	not calculated; see footnote
Race, N (%)					
White	107 (95.5)	<0.000	39 (34.8)	68 (60.7)	0.872
African American	5 (4.5)		2 (1.8)	3 (2.7)	

Table 2 shows patient comorbidities, including diabetes mellitus and hypertension, stage of chronic kidney disease, and diastolic dysfunction grade for the total population and grouped by physician-ordered UO monitoring. For the total population, statistically significant comorbidities (p<0.000) were hypertension (29 (25.9%)) and hypertension in combination with chronic kidney disease (21 (18.8%)), and hypertension in combination with chronic kidney disease and diabetes mellitus (30 (26.8%)), with the highest occurrence. There was no statistically significant difference in comorbidities between the groups based on UO monitoring. Of the patients with CKD, 49 (44.1%) had Stage 2 Mild CKD (p < 0.000). Diastolic dysfunction grade for the total population (p=0.321) and by group (p=0.599) was not statistically significant. Of note, 96 (85.7%) of patients included were treated with loop diuretics, which included furosemide, torsemide, bumetanide, and ethacrynic acid (p=0.000). 

**Table 2:** Patient comorbidities, chronic kidney disease, and diastolic dysfunction grade for the total population and grouped by physician ordered UO monitoring. KD: kidney disease; DM: diabetes mellitus; HTN: hypertension; CKD: chronic kidney disease; UO: urine output; GFR: glomerular filtration rate. * Chi-square test was performed to determine statistical differences between categorical variables. **Stage 1 defined as GFR>90mL/min (normal or high GFR). Stage 2 is defined as GFR=60-89 mL/min. Stage 4 is defined as GFR=15-29 mL/min. Stage 3A is defined as GFR=45-59 mL/min. Stage 3B is defined as GFR=30-44 mL/min. Grade I defined as E/A<0.8, avg E/e'<10, TR peak velocity<2.8m/s. Grade II is defined as E/A.8-2, avg E/e'10-14, TR pv>2.8m/s. Grade III is defined as E/A>2, avg E/e'>14.

Variable*	Total Population			Physician Ordered UO Monitoring						
				0=No		1=Yes		Total		P-Value
	Frequency	Percent	P-Value	Frequency	Percent	Frequency	Percent	Frequency	Percent	
Comorbidity										
0 = none	4	3.6	<0.000	1	0.9	3	2.7	4	3.6	0.64
1 = KD	5	4.5		1	0.9	4	3.6	5	4.5	
2 = DM	2	1.8		2	1.8	0	0	2	1.8	
3 = HTN	29	25.9		11	9.8	18	16.1	29	25.9	
4 = KD & DM	2	1.8		1	0.9	1	0.9	2	1.8	
5 = KD & HTN	21	18.8		9	8.0	12	10.7	21	18.8	
6 = DM & HTN	19	17		6	5.4	13	11.6	19	17	
7 = KD, HTN & DM	30	26.8		10	8.9	20	17.9	30	26.8	
KD**										
Stage 1 CKD	5	4.5	<0.000	1	0.9	4	3.6	5	4.5	0.094
Stage 2 Mild CKD	49	44.1		18	16.2	31	27.9	49	44.1	
Stage 3A Moderate CKD	27	24.3		6	5.4	21	18.9	27	24.3	
Stage 3B Moderate CKD	20	18		8	7.2	12	10.8	20	18.0	
Stage 4 Severe CKD	10	9		7	6.3	3	2.7	10	9.0	
Diastolic Dysfunction Grade										
Grade I Mitral	16	27.1	0.321	6	10.2	10	16.9	16	27.1	0.599
Grade II Mitral	25	42.4		12	20.3	13	22.0	25	42.4	
Grade III Mitral	18	30.5		6	10.2	12	20.3	18	30.5	

Table 3 demonstrates the main outcomes of the study. Of the total population, 84 (75.0%) patients did not have a readmission within 30 days (p<0.000). A total of 71 (63.4%) received urine monitoring every 8 hours (p<0.000), with 89 (79.5%) patients’ providers being private physicians rather than teaching service teams. (p<0.000). In addition, 87 (77.7%) patients did not receive a nephrology consult (p<0.000), and of those who did, 15 (13.4%) received the consult on day one of admission (p<0.000). 

**Table 3:** Association between clinical variables and physician ordered UO monitoring. PRN: pro re nata; UO: urine output. * Chi-square test was performed to determine statistical differences between categorical variables. An independent t-test was performed to compare the means of continuous variables. **  No p-value was calculated for physician-ordered monitoring because the frequency of urine monitoring per patient is a constant.

Variable*	Total Population		Physician Ordered UO Monitoring		
	Statistic	P-Value	No	Yes	p-Value
Length of Stay, mean days	6.09	NA	5.83	6.24	0.535
Readmission within 30 Days, percentage					
No	75.0	<0.000	68.3	78.9	0.213
Yes	25.0		31.7	21.1	
Frequency of Urine Monitoring per Patient, percentage**					
Every 8 hours	63.4	<0.000	0	63.4	not calculated; see footnote**
Once a day	29.6		0	29.6	
PRN	1.4		0	1.4	
Continuous	2.8		0	2.8	
QShift	2.8		0	2.8	
Nephrology Consulted, percentage					
No	77.7	<0.000	75.6	78.9	0.689
Yes	22.3		24.4	21.1	
Time of Nephrology Consult, percentage					
Day one of admission	13.4	<0.000	19.5	9.9	0.22
Later than day one	8.9		4.9	11.3	

Of the 112 patients reviewed, there were no statistically significant differences between physician-ordered UO monitoring based on mean LOS (p=0.535), readmission within 30 days (p=0.213), whether nephrology was consulted (p=0.689), and if consulted, the time of consult (p=0.22) (Table 3). The primary statistically significant result is that 71 (63.4%) of these non-ICU HF patients admitted did receive physician ordered monitoring of intake and urine output (p< 0.000) (Table 3). 

## Discussion

There is currently a lack of data discussing the benefit of monitoring UO in non-ICU patients with HF [24, 25]. UO is a surrogate marker of response to diuretics and can be used for dose adjustment. Even so, we discovered that LOS in these patients is not significantly affected by the physician's order to monitor intake and UO. This is clinically significant for several reasons. First, the decision to monitor UO may not be an effective use of resources. Second, in a non-ICU setting, UO monitoring may be less accurate with unmeasured or uncharted voids. Given the above and the non-significant correlation between UO monitoring and LOS in our study, this suggests that other methods of measuring diuresis may be more practical to obtain and therefore more helpful in monitoring effective management of HF exacerbation. For example, one study has shown that diuresis based on serial point-of-care ultrasound (POCUS) evaluations of the inferior vena cava and lungs had reduced the required days of in-hospital diuresis [29]. Since the LOS and readmission rates were not significantly affected, our results could suggest a trend that UO monitoring may be less effective in managing these patients. In fact, the mean LOS is slightly higher in patients with the physician's order to monitor UO (6.24 days) compared to patients without the order (5.83 days), though it should be noted that this finding was not statistically significant. 

Of the 112 patients, 49 (44.1%) had Stage 2 Mild CKD. Further studies are needed to investigate whether non-ICU HF patients with more severe stages of CKD would benefit from UO monitoring compared to those patients with milder stages of CKD. We also discovered that 84 (75%) of the patients were not readmitted within 30 days. Our results show that this lack of readmission is not due to physician-ordered UO monitoring. Future studies can investigate which variables are responsible for 30-day readmission rates in non-ICU HF patients.

An additional avenue used to estimate fluid status is body weight measurements. Past studies have proven that when body weight is accurately and routinely measured, weight gain in critically ill patients is a predictor of increased mortality [16, 17, 20]. Thus, there is data demonstrating the benefit of measuring body weight, specifically in ICU patients. However, maintaining accurate, daily measurements in routine clinical practice is widely variable. Furthermore, literature on the benefits of body weight measurements in non-ICU patients is limited. Further studies investigating the efficacy of body weight measurements in non-ICU patients may be beneficial in improving patient outcomes. 

There may be more effective measurements of resolution of HF exacerbation than volume status alone. As mentioned above, natriuresis-guided strategies have been shown to decrease hospital LOS, and this merits further investigation to provide more tailored therapy for each patient presenting with HF exacerbation [8]. 

Limitations of this study involve the characteristics of the patient sample. The mean age of included patients was 77 years old, 65 (58%) were female, and 107 (95.5%) were Caucasian (Table 1). 96 (85.7%) of patients were also on loop diuretics (furosemide, torsemide, bumetanide, and ethacrynic acid). The effects of UO monitoring may differ if the sample population included a larger proportion of younger patients or those on different classes of diuretics. Further, exclusion criteria aimed at reducing the number of patients not amenable to diuresis, such as those with ESRD on hemodialysis (HD) and those with anuria, may have removed renal patients where a nephrology consult would have taken a larger role in management and thus have a greater impact on length of stay. 

## Conclusions

Current AHA/ACC/HFSA guidelines recommend measurement of fluid intake/output in patients hospitalized with exacerbations of HF. There is little data directly linking UO monitoring with shortened LOS, specifically in non-ICU HF patients, a primary goal of our study. Our study showed that the physician's order to monitor urine output did not significantly affect LOS or readmission rates in non-ICU patients with exacerbation of HF. Additionally, consulting nephrology did not significantly affect LOS or readmission rates in these patients, though, as previously mentioned, those results may have been affected by the chosen exclusion criteria. Different methods in estimating volume status (e.g., daily body weight measurements, noninvasive monitoring devices) may substantially affect LOS, and future studies are needed.
